# Using Mobile Assessments to Characterize Mental and Physical Health Behaviors in Youth: Protocol for a Pilot Intensive Longitudinal Study

**DOI:** 10.2196/70990

**Published:** 2025-10-21

**Authors:** Konstantin Drexl, Sébastien Urben, Kerstin Jessica Plessen, Jennifer Glaus

**Affiliations:** 1 Division of Child and Adolescent Psychiatry, Department of Psychiatry, Lausanne University Hospital and University of Lausanne, Avenue d'Échallens 9, Lausanne, 1018, Switzerland, 41 213142475; 2 Clinical Psychology Unit for Developmental and Intellectual Disabilities, Faculty of Psychology and Educational Sciences, University of Geneva, Geneva, Switzerland

**Keywords:** mobile assessment, adolescents, intensive longitudinal study, feasibility, mental health, sleep, physical activity, dietary habits, health behaviors

## Abstract

**Background:**

Promoting healthy behaviors including adequate sleep, regular physical activity, balanced diet, and abstinence from substance use, alongside nurturing affective functioning, may crucially support physical and mental well-being in youth. Yet, little is known about how these domains interact dynamically in their daily lives.

**Objective:**

This pilot study evaluates the feasibility of prolonged, multiwave ambulatory assessment in school-recruited adolescents as well as in clinical and school-recruited preadolescents. In addition, it will examine the dynamic interplay of different health behavior domains and affective functioning in adolescent samples recruited in schools.

**Methods:**

The initial target sample size was 100 youths (ages 9‐17 years) across 2 assessment protocols. Adolescents complete three 3-week waves with 4 daily ecological momentary assessments and parallel actigraphy, and preadolescents follow two 10-day assessment waves. Feasibility aims will be assessed via equivalence tests targeting an a priori level of missing data and dropout at 70% each. Dynamic modeling in adolescent datasets will be addressed by a planned multilevel analysis leveraging idiographic effects as predictors of symptom outcomes, as well as state-of-the-art exploratory frameworks.

**Results:**

By July 2025, we enrolled 113 school adolescents and 27 school preadolescents exceeding the a priori target. Recruitment and data collection are still ongoing in the clinical preadolescent group of currently 13 preadolescents.

**Conclusions:**

This concise, intensive longitudinal protocol provides the basis for examining the feasibility of long-term intensive longitudinal paradigms in youth and lays a critical foundation for future large-scale studies aimed at unpacking the evolving interplay of health behaviors and emotional well-being.

## Introduction

### Addressing Health Behavior Domains in Youth Mental Health Research

Late childhood and adolescence represent critical developmental windows characterized by significant physiological, psychological, and social transformations. During this period, youth respond to these numerous biopsychosocial challenges with increasing personal autonomy, manifesting profound shifts across multiple health behavior patterns, including sleep [1-3], physical activity [4,5], dietary habits [6-8], and substance use [9]. Across these health behavior domains, research has made major advances in how health behaviors shape somatic health [10-14], but their role in the development of mental disorders during late childhood and adolescence remains poorly understood [15]. Nonetheless, the importance of modifiable health behaviors for mental health is gaining public awareness, and stakeholders are advocating their integration into public mental health strategies, including primary prevention [16], positive psychology [17,18], and nonpharmacological interventions for youth [19].

Extant research focused on the prospective and causal relationship of individual health behavior domains, including sleep, physical activity, dietary habits, and substance use, and regarding the onset and severity of diverse mental disorders, including depression, anxiety, bipolar disorder, and attention deficit hyperactivity disorder [20]. However, these isolated lines of research consistently show that the same behavior often relates to multiple disorders, and conversely, the same disorder associates with multiple behaviors. This lack of specificity limits our ability to infer which particular behavior underlies which symptom constellation [21]. In other words, 2 adolescents with the same health behavior profile at a single point of measurement may follow divergent clinical trajectories in terms of type and severity of symptoms over time. Such multifinality of risk factors underscores the need to move beyond domain-specific studies and toward integrative, intraindividual investigations that capture the temporal interplay of multiple behaviors and affective states within each person [21,22].

In this integrative approach, affective functioning, including momentary emotional reactions, mood fluctuations, and emotion-regulation processes [23], is particularly important, as it constitutes the critical interface linking health behaviors to the emergence of psychiatric symptoms. Taken together, these health behaviors and affective states form a dynamic regulatory system characterized by numerous pairwise interdependencies. For example, exercise improves sleep [24], which, in turn, affects activity levels [25] and eating preferences [26]. Emotions also influence physical activity [27,28] and sedentary behaviors [29], which subsequently impact eating behaviors, such as emotional eating [30,31]. Though these investigations remain limited to bivariate group-level associations. To capture the complexity of these interacting regulatory systems, research should adopt an intraindividual, multivariate paradigm that models complementary characteristics of their temporal dynamics [32-35]. Longitudinal studies confirm that adolescents exhibit distinct, person‐specific trajectories and intraindividual dynamics in sleep [36], physical activity profiles [37,38], eating habits [39,40], and affective functioning [41]. In sum, we posit that the interdependencies among these health behavior domains reflect dynamic, regulatory adjustment processes to life circumstances that are both specific to the individual and continuously evolving over time.

Providing evidence for the interrelated nature of these health behavior domains and their intraindividual dynamics is crucial for a holistic approach to youth health and requires advanced technical solutions for field research. Mobile digital technologies used for ambulatory assessments [42], such as actigraphy and frequently repeated self-reports collected via ecological momentary assessment (EMA; or experience sampling method), enable real-time monitoring of multiple health domains in naturalistic settings [43-45]. EMA has been widely used to track smoking, alcohol, and drug use [43], and emotional states [44] in youth, whereas actigraphy has been used primarily to assess patterns of sleep and, eventually, activity patterns [45]. Combining EMA and actigraphy allows investigating the temporal dynamic interactions between health domains over different timescales, for example, by investigating the temporal fluctuation of affective functioning and sleep disturbances from the daily [46] to the monthly timescale [47]. However, the inclusion of preadolescent children in EMA data collection depends on their access and autonomy in using a smartphone or similar connected mobile device. Moreover, the adequacy of this method remains subject to a normative ethical consideration regarding the inherent burden of intensive longitudinal designs as well as the role of parental involvement [48].

Taken together, to detect early signs of maladaptive health behaviors, etiological research should embrace a multivariate, intraindividual, and temporally sensitive approach. While large-scale epidemiological evidence requires significant resources, pilot studies can pave the way by examining the feasibility of intensive longitudinal protocols and unraveling the dynamic interplay between health domains and youth mental health.

### The Current Study

This study aims to pioneer future intensive longitudinal designs for epidemiological research by addressing both feasibility and across different variations of a modular intensive longitudinal paradigm, as well as preliminary investigations of the dynamic links between health behaviors and mental health among youths. Our methodological ambition was to flexibly adapt a multiwave multitimescale paradigm to different target populations by adapting the intensity of data collection as well as the composition and modality of assessments. First, adolescents recruited in schools will monitor their sleep, physical activity, dietary behavior, substance use, and affective functioning across three 3-week assessment waves over a timeframe of approximately 1 school year. This protocol will be investigated for both feasibility targets as well as preliminary findings in the dynamic interplay among the target domains and mental health.

Second, the developmental and clinical imperative to understand how health behaviors influence youth mental health highlights the need for intensive longitudinal studies in preadolescent age groups, especially as they undergo critical clinical transitions. However, the substantial burden of such designs raises questions about their feasibility in these settings. It even raises ethical challenges to potentially overburden vulnerable youth without prior ground for assuming the feasibility of the essential design characteristics. Therefore, we extended this pilot study with a reduced intensive longitudinal protocol in 2 preadolescent settings: school‐recruited participants and psychiatric patients transitioning from intensive outpatient treatment to follow‐up care. For both preadolescent groups, we reduced the protocol to 2 waves spanning 10 days each. Further group-specific adaptations are described in the respective protocol descriptions.

For both the adolescent and preadolescent protocol, feasibility outcomes focus on participation metrics of response compliance (ie, the percentage of all answered among all planned EMA surveys) and participant retention. We will test the hypothesis that both remain at an acceptable level (ie, both at 70% or above) and stable across the different groups and waves. Achieving the required sample sizes for the evaluation of these outcomes represents a prerequisite for the evaluation of these outcomes (see “Sample size rationale”). Consequently, the corresponding number of invited youth and schools and the acceptance rate provide information on the feasibility and efficiency of the recruitment. Not achieving the targeted sample sizes or rates of retention and compliance will therefore lead to thorough reconsideration of central design parameters. Irrespective of the obtained outcome, we will collect feedback on the subjective experience and opinion of participants and young partners to identify weaknesses and barriers of the protocols that can orient future modification of the design.

Beyond assessing feasibility, we will leverage our comprehensive adolescent protocol and use advanced statistical approaches specifically designed for intensive longitudinal data to examine further dimensions of the bidirectional dynamics linking health behaviors and mental health. By complementing feasibility-related findings with these analytical techniques, we aim to strengthen methodological frameworks and optimize both the design and implementation of future research. Insights gained from this pilot study will therefore guide and refine the scope of subsequent epidemiological cohort studies, ultimately enhancing our understanding of how health behaviors influence youth mental health trajectories.

## Methods

### Design

The design of this study closely aligns with the methods promoted by the mobile Motor Activity Consortium for Health (mMARCH), a collaborative international network coordinated by the National Institute of Mental Health, Intramural Research Program (ZIA MH002954), that was created to facilitate the coordination of procedures, analyses [49], and data sharing among research groups collecting mobile technology data. As an essential resource for intensive longitudinal research, the consortium provides a modular assessment framework that we followed to implement 2 age-specific assessment protocols, one for adolescents and one for preadolescents. The study is currently in data collection and continues to recruit participants. This study protocol for a pilot intensive longitudinal investigation follows the reporting guidelines for cohort studies as required by the Strengthening the Reporting of Observational Studies in Epidemiology (STROBE) Statement [50] as well as the adapted STROBE Checklist for Reporting Ecological Momentary Assessment Studies [51] (CREMAS). The following sections will introduce the methods for the adolescent and the preadolescent protocol separately. For the entire project, the initial goal was to enroll a total of 100 participants from the French-speaking region of Switzerland. Enrollment started in November 2022, and data collection will end in September 2025. Each assessment protocol is adapted and implemented to best meet the respective educational or clinical context, while respecting the capacity of each age group to engage in an intensive longitudinal study.

### Adolescent Protocol

#### Participants

For the adolescent protocol, we planned to recruit 60 participants between the ages of 14 and 17 years from 4 private secondary and high schools. All schools opted for delivering invitations to the entire cohort meeting the respective age range. To this end, the research team held presentations, either during class sessions or in school assemblies.

#### Eligibility

Inclusion criteria are as follows: adolescents must be in possession of a smartphone and participants are able to speak French or English. We will exclude participants with severe motor or cognitive disability that involves deficits in core activities of daily living, and participants with a disorder of intellectual development or with a potential developmental learning disorder.

#### Procedures

**Figure 1.**
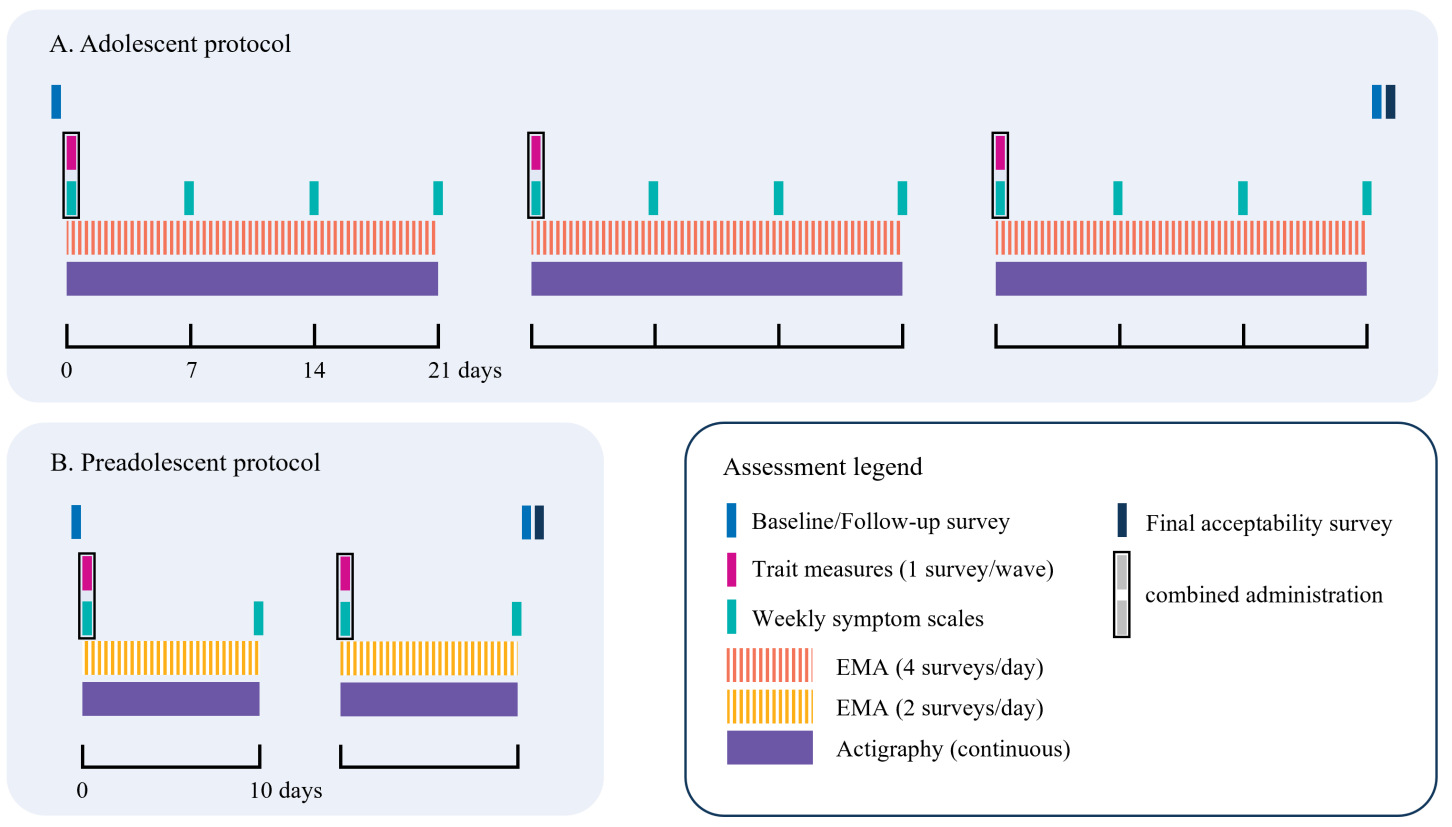
Timeline of assessment protocols. EMA: ecological momentary assessment.

An overview of the planned data collection timeline, following the guidelines recommended by the Standard Protocol Items: Recommendations for Interventional Trials (SPIRIT) [52] is presented in Figure 1 and Table 1. Participants will complete a baseline evaluation, followed by repeated waves of parallel EMA and actigraphy monitoring. The adolescent protocol consists of 3 waves, with each wave lasting 3 weeks. In addition, each assessment wave is accompanied by periodically repeating retrospective questionnaires, administered at the beginning of each period, as well as on a weekly basis. Finally, at the end of the study, participants will respond to a final psychopathological follow-up and an assessment of study acceptability and measurement reactivity. The protocol ends with personalized feedback delivered during group in-class sessions. All self-report measures, including EMA surveys, will be administered online via personal access links transmitted via SMS or email using REDCap (Research Electronic Data Capture; Vanderbilt University) [53,54]. All participants receive training sessions with live demonstrations of the EMA procedure in small groups. To prevent missing data due to technical failure or silent dropouts, the study team is contacting participants approaching the two-thirds limit to assist with any technical issues and remind them of the study protocol and its rationale.

**Table 1.** Multi-informant assessment plan for the participants by group.

Occasions	Adolescents	Preadolescents		
	All self-reported	Participant interview	Self-reported	Reported by the parent
Baseline	Demographic PPS^a^ CRISIS^b^ FERS-C^c^ MESC^d^ YSR^e^ PEARLS^f^-TEEN FFQ^g^	Demographic PPS CRISIS FERS-C MESC	CBCL^h^ PEARLS-CHILD FFQ
Start of each wave	SDQ^i^-social PAQ-A^j^ ESEA^k^	SDQ-social PAQ-C ESEA	—^l^	—
Weekly^m^	CES-DC^n^ STAIC^p^	CES-DC^o^ STAIC^o^	—	—
EMA^q^	Morning Noon Afternoon Evening	—	Morning Evening	—
Follow-up	YSR	—	—	CBCL
Round-up session	Acceptability+ Reactivity	—	Acceptability+ Reactivity	—

a PPS: Peterson Puberty Scale.

b CRISIS: Coronavirus Health Impact Survey.

c FERS-C: French version of Emotional Regulation Scale for Children.

d MESC: Morningness-Eveningness Scale for Children.

e YSR: Youth Self-Report.

f PEARLS: Pediatrics Adverse Childhood Events and related life events screener.

g FFQ: Food Frequency Questionnaire.

h CBCL: Child Behavior Checklist.

i SDQ: Strengths and Difficulties Questionnaire.

j PAQ: Physical Activity Questionnaire.

k ESEA: Sleepiness Scale.

l Not applicable.

m The first weekly assessment of each wave is included in the initial assessment battery of each wave.

n CES-DC: Center for Epidemiological Studies Depression Scale for Children.

o Preadolescent participants in schools self-report the weekly assessment at the end of their 10-day wave.

p STAIC: State-Trait Anxiety Inventory for Children.

q EMA: ecological momentary assessment.

Adolescent participants (Figure 1A) engage in three 3-week ambulatory assessment waves with 4 daily EMA surveys. Preadolescent participants (Figure 1B) take part in two 10-day waves with 2 daily EMA surveys. Intervals between waves range from 2 to 3 months depending on the school calendar. The initial trait measure and weekly symptom assessment of each wave are administered as one combined survey.

#### EMA

**Figure 2.**
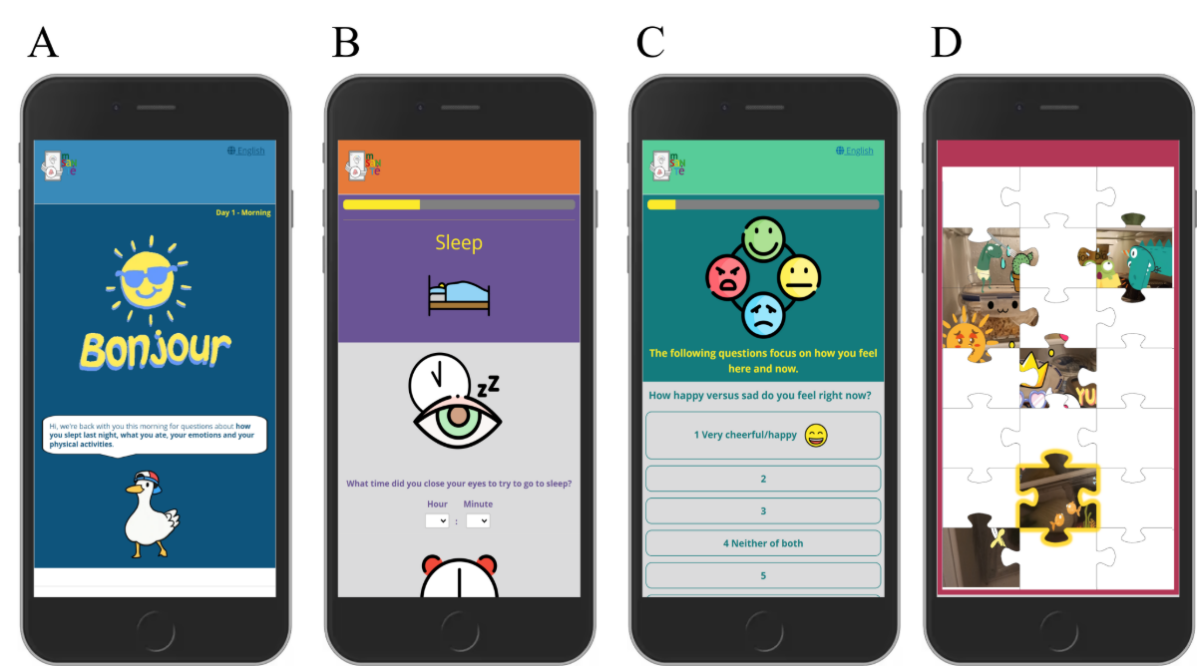
Screenshots of ecological momentary assessment surveys.

Adolescents receive 4 EMA surveys per day on their personal smartphone. Fixed delivery times are adapted to the respective schedule of each school to deliver surveys (1) on mornings before class (eg, 07:45 AM), (2) during lunch break (eg, 12 PM), (3) after the last class in the afternoon (eg, 3:45 PM), and (4) on evenings (eg, 7:30 PM). In case of nonresponse, participants receive 2 reminders in intervals of 20 minutes, and surveys remain accessible for either 3 hours for the first 3 surveys and 4 hours for the evening survey of each day. The design of the EMA surveys includes the choice of an individual avatar who will guide the participant through his or her “lifestyle journal,” as well as a gamified puzzle presented each evening to provide feedback on the progress and approximate compliance with their EMA schedule (Figure 2).

In Figure 2, example screenshots showing the welcome screen of the morning survey with the participant-chosen avatar (picture A), items on sleep timing (picture B), mood items (picture C), and the gamified compliance puzzle (21 pieces across 21 days of the adolescent protocol, picture D).

#### Actigraphy Recordings

During the whole period of each assessment wave, participants are continuously wearing a triaxial accelerometer (GENEActiv, Activinsights Ltd) around their nondominant wrist. Recordings are configured with a 30 Hz sampling frequency starting the afternoon preceding the first EMA morning delivery. Data are downloaded using the manufacturer’s software. Preprocessing will be conducted using the GGIR package [49,55,56] in R (R Core Team) and the pyActigraphy package [57] in Python to obtain readouts of physical activity and sleep patterns as implemented by the respective software.

#### Self-Report Measures

As part of our rationale to cover the dynamic interplay of health behavior domains and mental health, we implemented measures assessing target constructs across different timeframes spanning from static characteristics (eg, sex assigned at birth) over malleable traits (eg, depressive symptom severity and social functioning) to momentary states (eg, sad vs happy mood). The questionnaires are therefore administered and repeated in groups of baseline measures, wave-level measures, weekly symptom monitoring during each wave (Table 1), and EMA surveys (Table 2; Table S1 in Multimedia Appendix 1). For all measures, we sought psychometrically validated scales that are available in French and English whenever possible, favoring measures used by previous data collections of the mMARCH consortium [45,58]. We also considered brevity of instruments, especially for those scales that were added for this study. Two measures, the Pediatrics Adverse Childhood Experiences (ACEs) and related life events screener for parents of children (PEARLS-CHILD) and for teenagers (PEARLS-TEEN) [59], and the Physical Activity Questionnaire for Older Children (PAQ-C) and for Adolescents (PAQ-A) [60,61] were forward translated by 2 blinded members of the research team and synthesized in discussions supervised by a senior researcher.

**Table 2.** Ecological momentary assessment measures.

Section	Range of number of items
Sleep^a^	4
Activities and context	4‐6
Emotional and somatic states	12
Beverage intake	1‐11
Food intake	1‐5
Substance use^b^	1‐9
Physical activity	1‐2
Social media use^b^^,c^	2
Daily major events and emotion regulation^c^	10‐11
Physical health^c^	3‐6

a Only administered at the first active response of each day.

b Only administered to adolescent participants (Group 3, 14‐17 years).

c Only administered in the evening survey.

##### Baseline and Follow-Up Measures

Baseline assessments mainly serve for descriptive purposes and between-person covariates in longitudinal analyses. Instruments are presented in the order they are administered to the participants.

First, participants report on sociodemographic characteristics (eg, gender, age, and family composition) and their medical history (eg, presence of chronic disease, past surgical interventions, and psychiatric treatment) with items adapted from the CoLaus study [58]. To operationalize an indicator of pubertal maturation, we administer the Peterson Puberty Scale (PPS) [62]. Worries related to a COVID-19 infection of the participants themselves or of significant others are assessed by the COVID-19 worries subscale Coronavirus Health Impact Survey (CRISIS) based on 5 items [63]. Participants further rate their habitual emotional awareness, interpretation effort, and reactions based on the 15 items of the French Emotional Regulation Scale for Children (FERS-C), with English item wordings made available through the published validation study [64]. Chronotype represents a crucial bioregulatory trait for the target domains of this study [65]. We, therefore, assessed the preference of being more active and performant at earlier versus later hours using the 10 items of the Morningness-Eveningness Scale for Children (MESC) [2,66].

To examine a dimensional psychopathological profile across participants, we use the Youth Self-Report (YSR) of the Achenbach System of Empirically Based Assessment (ASEBA) [67,68]. This measure is at the end-of-study follow-up, allowing us to relate the within-person dynamics measured across the full study period to changes in broadband symptom severity. The YSR operationalizes psychopathology across a set of 119 behavioral and emotional problems covering 8 different dimensions: anxious-depressed, withdrawn-depressed, somatic complaints, social problems, thought problems, attention problems, rule-breaking behavior, and aggressive behavior.

Addressing a putative transdiagnostic risk factor, the PEARLS-TEEN [59] inquires about the presence of 10 potential traumatic events, such as abuse, neglect, and family violence. As the last measure of the baseline assessment, a Food Frequency Questionnaire (FFQ) specifically developed for the Swiss population of the Lemanic region [69,70] provides a past-week food intake recall across a wide range of 97 food and beverage items. Taken together, the reported frequencies allow estimation of latent dietary profiles (eg, Mediterranean diet) among the sample. The final end-of-study survey inquiring about study acceptability and measurement reactivity was adapted from a previously published feasibility study [71].

##### Wave-Level Measures

At the beginning of each assessment wave, participants provide a retrospective report on malleable traits we consider susceptible to change across periods of multiple weeks to multiple months. As part of this “moving baseline” for each assessment wave, participants fill the prosocial subscale of the Strengths and Difficulties Questionnaire (SDQ) [72-74]. This scale operationalizes social functioning indicated by 5 items on prosocial efforts, attitudes, and empathy. In addition, participants report habitual daytime sleepiness using the French Sleepiness Scale adapted to children and adolescents (Échelle de Somnolence adaptée à l’Enfant et à l’Adolescent [Somnolence Scale for Children and Adolescents]; ESEA) [75]. More specifically, respondents estimate the chances of dozing off or falling asleep across 10 different example activities.

In addition, participants are asked to recall their physical activity and exercise during the past 7 days based on the PAQ-A [60,61,76]. This measure incorporates a list of popular sports disciplines among youth, which we adapted to match the disciplines favored by a representative Swiss sample of adolescents [75].

##### Weekly Measures

Each assessment wave is accompanied by weekly monitoring of depressive and anxiety symptoms, allowing us to relate momentary fluctuations in mood and health behaviors to changes in retrospective self-reports. The Center for Epidemiological Studies Depression Scale for Children (CES-DC) assesses depressive symptoms [77-79], and the State-Trait Anxiety Inventory for Children (STAIC) [80,81] inquires symptoms of anxiety, both scales consisting of 20 items each.

##### EMA Surveys

Items incorporated in the EMA protocol were selected to balance multiple considerations, including the broad scope of the study, survey duration, sensitivity to youth-specific processes, and, finally, relevance for local youth. First, we adapted a subset of the item catalog harmonized by the mMARCH consortium [82]. Second, we included 2 additional items on emotional overload and loss of control used in a prior project [83]. Third, 7 items assessing distinct emotion-regulation strategies (eg, reappraisal and distraction) were adapted from another published study [84]. Finally, the substance use section (including vaping for both nicotine and tetrahydrocannabinol) was tailored to local adolescent consumption patterns. The resulting item catalog yields a technical range of 20‐68 items depending on skip-logic (see Table 2 for an overview of sections and Multimedia Appendix 1 for the full EMA item catalog). Preliminary piloting resulted in response durations between 2 and 7 minutes maximum.

### Participant Involvement

Participants from all groups are invited to feedback sessions at the end of the study, giving them the opportunity to express their honest opinion about the study. In addition, we will invite motivated adolescent participants to become young partners and contribute to a dedicated in-depth discussion session on the research project.

### Preadolescent Protocol

Our presentation of the preadolescent protocol primarily concentrates on differences relative to the adolescent protocol and eventual differences between the school-recruited and clinical subgroups among preadolescent participants. Design characteristics that are not explicitly specified for preadolescents align with the description of the adolescent protocol.

#### Participants

For the preadolescent protocol, we intended to include 20 preadolescents between the ages of 10 and 13 years from private secondary schools, as well as 20 preadolescent patients between the ages of 9 and 12 years from an intensive outpatient treatment program. Patients commonly present with behavioral disturbances (eg, oppositional defiant behavior and emotion-regulation difficulties), communication or language disorders (eg, speech delays), specific learning disorders (eg, reading or writing impairments), and socialization problems (eg, limited peer interactions). Patients and their families receive intensive psychiatric care to sustain the psycho-affective and social development of the patient and to construct a therapeutic project for ambulatory follow-up care. Recruitment of eligible preadolescent patients is organized on an individual basis and is still ongoing.

#### Eligibility

Most eligibility criteria of the adolescent protocol also apply to the preadolescent protocol. The sole difference consists of preadolescents having to have access to a smartphone of one of their parents or legal representatives instead of being in possession of their own smartphone.

#### Procedures

The preadolescent protocol entails 2 waves of parallel EMA and actigraphy monitoring, with each wave lasting 10 days. During each ambulatory assessment wave, preadolescent participants respond to 2 EMA surveys per day (morning and evening) via their parents’ smartphones. Morning deliveries are scheduled individually to allow families to be at the same place before heading off to school or work. Preadolescent participants recruited from schools receive EMA training in small groups, whereas clinical participants and their parents receive individual training. Instead of repeating retrospective symptom reports at exactly 7-day intervals as in the adolescent protocol, preadolescents respond to these measures at a 10-day interval, once at the beginning and end of each assessment wave. Actigraphy recordings are configured as described in the adolescent protocol, with recordings interrupted at the end of the 10-day period upon return of the device.

#### Participant Involvement

The design adaptations for the clinical preadolescent group were informed by discussions with parents of the clinical target population. This feedback was particularly influential in the design of the preadolescent clinical protocol. Participants recruited in the school setting will be invited to feedback sessions, whereas participants from the clinical setting will be invited to individual feedback sessions with their parents.

#### Self-Reported, Parent-Reported, and Clinician-Administered Measures

To reduce participation burden for preadolescent participants, we adapted the specific choice of instruments and modality of data collection (Table 1) to best meet their capacities of needs as well as those of their parents.

##### Baseline and Follow-Up Measures

During baseline assessments, the majority of instruments, more specifically the sociodemographic and medical history instruments [58], as well as the PPS [62], CRISIS [63], and FERS-C [64], are administered in an interview setting. Baseline self-reports from preadolescents were limited to the MESC [2,66], which uses adequate language for this age group (eg, “Imagine school is canceled. You can get up whenever you want to. When would you get out of bed?”). Parents responded to the respective parent-report versions for the general psychopathology assessment by means of the ASEBA Child Behavior Checklist (CBCL) [67,68] followed by the parent-report version of the ACEs screener (PEARLS-CHILD) [59]. Parents also report on baseline dietary habits of their participating child (FFQ) [69,70], with alcoholic beverage items omitted for this age group. Consistent with the follow-up planned for adolescent participants, parents repeat the CBCL at the end of the protocol, while their children respond to the final questions on study acceptability and measure reactivity.

##### Wave-Level and Weekly Measures

At the beginning of each wave, preadolescent participants respond to the retrospective assessments of social functioning (SDQ-social) [72-74], daytime sleepiness (ESEA) [75], and physical activity in an interview setting. For the physical activity questionnaire, a dedicated children’s version was available (PAQ-C) [60,61,76]. Retrospective self-reports of symptoms of depression (CES-DC) [77-79] and anxiety (STAIC) [80,81] were administered in self-report format for school-recruited preadolescents and in interview format for clinical participants, respectively.

##### EMA Surveys

Preadolescents complete a reduced set of EMA items compared to adolescent participants (Table 2; Multimedia Appendix 1). Due to the negligible likelihood of psychotropic substance exposure in this age group [85], we exclude items related to substance use in their EMA prompts. Similarly, questions concerning alcohol consumption are omitted from the EMA surveys for preadolescents. In addition, the item addressing current menstruation is presented exclusively to female participants who report having experienced the start of menarche at baseline or at the onset of the second assessment wave.

### Statistical Analyses

The targeted feasibility outcomes of acceptable and stable participation metrics will be examined by appropriate tests of equivalence regarding compliance and dropout against a priori benchmarks for both protocols [86]. We will further examine the stability of participation metrics by means of separate equivalence tests. We will also explore potential determinants of missing data and problematic response behaviors such as careless responding that might be clinically informative and that span different timescales as we test predictions from the retrospective baseline level (eg, ACEs) to the momentary level (eg, sedentary behavior).

To comprehensively address the inherent temporal and mechanistic complexity of dynamic interactions among health behaviors and affective functioning, multiple complementary statistical frameworks will be applied to data from the adolescent protocol. Each analytical model targets distinct characteristics of this interplay. We coregistered a first set of hypotheses claiming substantial heterogeneity in the daily within-person couplings of sleep patterns and daytime mood among adolescents [75,76]. Building upon prior research on internalizing etiology [87,88], we further hypothesize that the intensity of mood-sleep couplings is associated with levels of internalizing symptoms within the subclinical spectrum. These analyses apply Bayesian multilevel modeling [89,90].

To further contrast the relation between interindividual and intraindividual variability, time-series data will be analyzed to identify average and individual variability in motor activity, sleep patterns, eating habits, and associations with emotional states over different time periods (1 vs 2 vs 3 weeks; time of the day; day of the week; month and season). In order to provide preliminary findings on the different dynamic features of the interplay between health behaviors and mental health in youth, stability of sleep, physical activity, eating, and emotional states and their interrelationships will be assessed via different statistical approaches: (1) fragmentation analysis will be used to quantify subject-specific stability [91]; (2) functional principal component analyses and other machine learning approaches, such as the Joint and Individual Variance Explained (JIVE) [92] will be applied on the rich actigraphy dataset to explore the key components of sleep and physical activity, including average intensity, variability, and timing [93]; (3) Generalized Estimating Equations and network modeling [94] will be used to test the conditional estimate of the stationary dynamic associations between sleep, physical activity, eating, and emotional states, incorporating the lagged associations between these domains. Collectively, these approaches provide complementary rather than competing perspectives to deepen understanding of the dynamic interrelationships between health behaviors and mental health in youth.

Psychometric properties of the in-house translated scales (ie, PEARLS-CHILD, PEARLS-TEEN, PAQ-C, and PAQ-A) will be assessed based on internal consistency and construct validity. We will examine criterion validity by correlating ACEs with general psychopathology and self-reported physical activity with objectively recorded actigraphy. In addition, we will assess the test-retest reliability of the PAQ-C and PAQ-A scales across the 3 assessment waves based on changes observed in actimetry recordings.

### Sample Size Rationale

Regarding the mixed populations and protocols within the present pilot study, we plan to balance data availability and statistical robustness for our feasibility targets and within-person effects. With respect to the limited annual intake of patients in the outpatient treatment who are eligible for group 1 (approximately 15 per year), our intention was to test feasibility per protocol with the combined preadolescent group reaching a sample size of 40 (ie, 20 each). Targeting the feasibility of the present multiwave design, we calculated statistical power for frequentist equivalence tests supporting the stability of response compliance rates across assessment waves. Based on equivalence bounds covering change scores within 70%±10%, a nominal alpha level of .05, and modest variability in change of response compliance (SD 0.1), statistical power of results in favor of stable compliance rates was considered adequate for the preadolescent protocol (power=0.87, n=40). This was also the case for the adolescent protocol (power=0.97, n=60), even under conservative Bonferroni correction accounting for 3 wave-specific equivalence tests (α=.015, power=0.91; see Figure S1 in Multimedia Appendix 1).

The rationale for determining sample size in the adolescent protocol, with regard to registered Bayesian multilevel models, was based on a reliability metric [95] for within-person effects between sleep patterns and daytime mood. A detailed explanation of this precision-oriented sample size calculation is available in the openly registered analysis plan [96]. Of note, precision-oriented determination of sample size aligns with principles of Bayesian analysis, emphasizing the accuracy of parameter estimates when comparing evidence for competing hypotheses [97].

### Missing Data Handling

Given the intensive longitudinal design and the likelihood of both intermittent and monotone missingness, our complementary analytic approaches will use listwise deletion or full-information maximum likelihood estimation to accommodate unbalanced data under a missing-at-random assumption. Where missingness may be informative, such as dropout being related to symptom severity, school holidays, or duration of participation, we aim to extend these models to jointly estimate the missingness mechanism and outcome trajectories using selection models within a structural equation modeling framework [98] or via joint multilevel survival models [99]. For variables exhibiting sporadic missingness under missing-at-random, we will perform multiple imputation by chained equations, generating at least 20 datasets. Finally, sensitivity analyses will compare these results to complete-case analyses to evaluate the robustness of our findings.

### Ethical Considerations

The study was approved by the Ethics Committee of the Vaud Canton, Switzerland (Project-ID 2021‐01821) and is in accordance with the Declaration of Helsinki. All participants received oral and written information about the study and received enough time to ask any questions. Adolescent participants provided written consent. Parents of adolescent participants received written information and were invited to reach out to the research team in case of any questions. Parents of preadolescent patients are met in person to explain the study and provide information letters and informed consent forms, whereas participants recruited in schools are asked to transmit information letters to their parents and return signed forms to the school staff or to the research team. Parents of both groups were encouraged to ask any questions regarding the study.

Adolescent participants will receive gift vouchers at a technology and culture retail chain of CHF 30 (US $37.72) at the end of the first and second, and CHF 40 (US $50.29) at the end of the third wave, respectively, when completing at least two-thirds of the self-report measures. Preadolescent participants receive gift vouchers of CHF 20 (US $25.15) and CHF 30 (US $37.72) at a technology and culture retail chain at the end of each respective wave, when completing at least two-thirds of the self-report measures.

During the ethics review, the local ethics committee recommended that such exclusion be avoided, noting that it could be perceived as unfair by students who had shown interest in participating. Participating schools opted to present the study to entire age cohorts, making oversampling a foreseeable outcome. This approach was formally documented in an amendment and received prior ethical approval.

## Results

As of July 2025, we enrolled 113 adolescent and 27 preadolescent students, 13 preadolescent patients from the outpatient unit. The overrecruitment observed in the school-based samples, particularly among adolescents, reflects our ethical consideration to avoid excluding students solely based on the achievement of the predefined sample size.

Recruitment and data collection are still ongoing in the clinical setting. Every group in the school setting received feedback sessions with dedicated time to discuss the participants’ experience of being involved in the study. Furthermore, we held one participatory session with 4 adolescent students from one school to discuss the strengths and limitations of the study in more detail.

## Discussion

### Principal Research Targets

This research highlights and attempts to capture the developmental dynamics of mental and physical health during emerging adolescence, using assessments across multiple timescales for in-depth, longitudinal analysis. Our feasibility-related findings will be crucially informative for future field research, especially in epidemiological paradigms. By examining the complex interplay between health behaviors across varying stages of late childhood and adolescence, we will gain preliminary insights into the transition from healthy states to those with mental health problems. This approach is pivotal for promoting health literacy, for developing age-appropriate early detection and prevention strategies, including transdiagnostic interventions in community and in clinical settings [100]. Using mobile technologies enables the creation of school-based programs for promoting healthy behaviors and discreetly identifying at-risk youth, particularly in older children and adolescents before the onset of many psychiatric disorders [101]. Because the study of these multiple health domains in youth using methods such as EMA and actigraphy is still struggling to converge on coherent frameworks of developmental psychopathology [102], the present methodological groundwork is essential across different clinical contexts and age groups.

### Limitations

Despite its commendable attributes, notably the rich and multifaceted longitudinal data, this research project has inherent limitations that need to be addressed. First, clinical participants were drawn exclusively from a single intensive outpatient treatment unit for preadolescents, and school‐recruited youth attended private institutions, resulting in unbalanced and unrepresentative sampling, which impedes group comparisons. Populations recruited from private schools likely differ systematically from public schools and community clinics in socioeconomic profile [103], access to beneficial environments (eg, parental support and calm and safe sleep environment) [104], and development of health behaviors [105]. For example, private‐school adolescents, who typically come from higher socioeconomic status (SES), have been shown to select healthier snacks and consume more fruits and vegetables than their public‐school peers [106], and high‐SES adolescents generally engage in greater levels of physical activity compared to lower‐SES youth [107]. Conversely, clinical patients represent a subgroup characterized by greater symptom severity and comorbidity, with adolescents accessing specialty mental health services tending to have more severe and persistent disorders than those in community settings [108]. Consequently, the temporal dynamics of sleep, activity, diet, and mood we characterize may not extend to adolescents in lower‐resource environments, public school settings, or clinical engagement. Our unbalanced design impeded corresponding group comparisons. Future large‐scale studies should purposively sample across diverse sociodemographic and educational contexts, including public schools, community mental health centers, and underresourced regions, to evaluate whether the longitudinal dynamics we observe hold across the broader population of youth. Second, while we aimed for a comprehensive evaluation of relevant health behavior domains, some areas may have been missed, such as interpersonal relations and peer dynamics. Third, the lack of detailed data on stressors and significant life events such as relationship break-ups or familial loss during the study is a limitation. Anticipated events, such as final school examinations, were also not examined. Fourth, the translation of selected questionnaires without formal validation due to the multilingual study cohort may be a limitation. Fifth, the reliance on self-reported measures, including both retrospective trait questionnaires and in situ EMA surveys, introduces potential biases such as recall error [109], social desirability [110], and measurement reactivity, which may affect the accuracy and generalizability of our findings. Although EMA minimizes recall intervals [111], participants’ awareness of ongoing monitoring can alter their responses. Future work should consider the most direct and unbiased assessment modalities available or under development (eg, passive dietary sensors and ecological smartphone usage logs) [102] or multimethod approaches to mitigate these self-report biases.

Despite these limitations, the comprehensive exploration of key health behavior domains and mental health in developing youth holds promise as a foundation for future studies. These findings can inform interventions and training programs to enhance prevention and intervention strategies for individuals.

### Clinical and Research Implications

The present multimodal ambulatory monitoring paradigm can be seamlessly embedded within routine therapeutic care by choosing the specific domain most relevant for a given clinical situation. At treatment initiation, EMA and actigraphy can be used collaboratively by patient and clinician to coconstruct a personalized case formulation, providing an “additional eye” on day-to-day symptom dynamics and functional patterns [112,113]. By repeating these assessments at midtherapy and during key care transitions (eg, stepping down from intensive support), clinicians and patients can discuss their perceptions in front of the changes captured between sessions to refine formulations, adjust treatment targets, and evaluate response in real time [6, 114,115].

Going forward, research should thoroughly test the validity of temporal and digital markers that emerge from fundamental psychopathology studies and translate into practical, in-session tools [113]. Such work will determine which patterns of sleep, activity, stress reactivity, or digital engagement most reliably map onto clinical processes (eg, remission and relapse) and other consequential outcomes, and how to present these markers to clinicians [116]. Ultimately, this line of research will bridge ambulatory assessment science and everyday practice, enhancing transparency, engagement, and, hopefully, the effectiveness of youth mental health interventions.

### Conclusions

This research aims to strengthen the investigation of the intricate dynamics between mental and physical health during adolescence by investigating the interplay between health behaviors and affective functioning across multiple timescales. The findings will not only contribute to methodological advancements but also inform future large-scale epidemiological studies and interventions aimed at early detection and prevention of mental health issues, highlighting the importance of idiographic approaches in youth health research.

## Supplementary material

10.2196/70990 Multimedia Appendix 1 Ecological momentary assessment items and power curves.

10.2196/70990 Checklist 1 STROBE checklist.

10.2196/70990 Checklist 2 CREMAS checklist.

## Abbreviations

ACE: Adverse Childhood Experiences
ASEBA: Achenbach System of Empirically Based Assessment
CBCL: Child Behavior Checklist
CES-DC: Center for Epidemiological Studies Depression Scale for Children
CREMAS: Checklist for Reporting Ecological Momentary Assessment Studies
CRISIS: Coronavirus Health Impact Survey
EMA: Ecological Momentary Assessment
ESEA: Échelle de somnolence chez l’enfant et l’adolescent [Sleepiness Scale]
FERS-C: French version of Emotional Regulation Scale for Children
FFQ: Food Frequency Questionnaire
MESC: Morningness Eveningness Scale for Children
mMARCH: Mobile Motor Activity Consortium for Health
PAQ-A: Physical Activity Questionnaire for Adolescents
PAQ-C: Physical Activity Questionnaire for Older Children
PEARL-TEEN: Pediatrics Adverse Childhood Events and related life events screener for teenagers
PEARLS-CHILD: Pediatrics Adverse Childhood Events and related life events screener for parents of children
PPS: Peterson Puberty Scale
REDCap: Research Electronic Data Capture
SDQ: Strengths and Difficulties Questionnaire
SES: socioeconomic status
SPIRIT: Standard Protocol Items: Recommendations for Interventional Trials
STAIC: State-Trait Anxiety Inventory for Children
STROBE: Strengthening the Reporting of Observational Studies in Epidemiology
YSR: Youth Self-Report
