# Supplementary material for: Using Mobile Assessments to Characterize Mental and Physical Health Behaviors in Youth: Protocol for a Pilot Intensive Longitudinal Study
Source: JMIR Res Protoc. 2025 Oct 21;14:e70990. doi: 10.2196/70990 (PMC12539651; doi:10.2196/70990)
Supplement: Multimedia Appendix 1 [file resprot-v14-e70990-s001.pdf]

This is a Multimedia Appendix to a full manuscript published in the J Med Internet Res. For full copyright and citation information see http://dx.doi.org/10.2196/jmir.xxxx

Drexl K[a], Urben S[a], Plessen KJ[a], Glaus J[a]

[a]Division of Child and Adolescent Psychiatry, Department of Psychiatry, **Lausanne University Hospital and University of Lausanne**, Lausanne, Switzerland

Table S1. EMA surveys

**Questions asked once in the morning**	
**Sleep**	
What time did you close your eyes to try to go to sleep?	Hour Minute<br>____ : ____
What time did you wake up this morning?	Hour Minute<br>____ : ____
How would you rate the quality of your sleep?	1◯ 1 very poor<br>2◯ 2<br>3◯ 3<br>4◯ 4 medium<br>5◯ 5<br>6◯ 6<br>7◯ 7 very good
How refreshed did you feel when you woke up?	1◯ 1 not at all refreshed<br>2◯ 2<br>3◯ 3<br>4◯ 4 moderately refreshed<br>5◯ 5<br>6◯ 6<br>7◯ 7 fully refreshed
**Questions asked four times a day (or 2 times a day for preadolescents)**	
**Context**	
Where are you right now?	1◯ In my home<br>2◯ In home of relative or friend<br>3◯ In class or at work<br>4◯ In a restaurant/cafe/bar<br>5◯ In a store or shop<br>6◯ In the gym or fitness center

	7◯ In a hospital or doctor's office<br>8◯ In a vehicle (car/bus/etc.)<br>9◯ In a public building<br>10◯ In a park or garden<br>11◯ Other place inside<br>12◯ Other place outside
Are you with someone right now?	1◯ Yes<br>2◯ No
[Only if yes]<br><br>Who is with you at this moment?	1☐ Family member<br>2☐ Partner/boyfriend/girlfriend<br>3☐ Friend<br>4☐ Colleague or classmate<br>5☐ Stranger<br>6☐ A pet (dog, cat...)<br>7☐ Other: _________
What are you doing at this moment?	1☐ Nothing or waiting<br>2☐ Napping/resting<br>3☐ Eating<br>4☐ Household chores<br>5☐ Shopping<br>6☐ Personal hygiene care<br>7☐ Physical leisure or sports<br>8☐ Walking the dog<br>9☐ Traveling or commuting<br>10☐ Listening to music<br>11☐ Watching TV<br>12☐ Use social media<br>13☐ Talking on the phone<br>14☐ Talking in person<br>15☐ Working (paid or volunteer)<br>16☐ Other nonphysical leisure<br>17☐ Other activity: __________
The following questions focus on how you feel here and now.	
How happy versus sad do you feel right now?	1◯ 1 Very cheerful/happy<br>2◯ 2<br>3◯ 3<br>4◯ 4 Neither of both<br>5◯ 5<br>6◯ 6<br>7◯ 7 Very sad/depressed/unhappy
How relaxed versus anxious do you feel right now?	1◯ 1 Very relaxed/calm<br>2◯ 2<br>3◯ 3

	4◯ 4 Neither of both<br>5◯ 5<br>6◯ 6<br>7◯ 7 Very nervous/anxious

How calm vs. excited do you feel right now?	1◯ 1 Calm/quiet<br>2◯ 2<br>3◯ 3<br>4◯ 4 Moderately excited/aroused<br>5◯ 5<br>6◯ 6<br>7◯ 7 Very excited/aroused
How tired vs. energetic do you feel right now?	1◯ 1 Very tired/sluggish<br>2◯ 2<br>3◯ 3<br>4◯ 4 Neither of both<br>5◯ 5<br>6◯ 6<br>7◯ 7 Very energetic/lively
How well can you concentrate or focus right now?	1◯ 1 Very focused/attentive<br>2◯ 2<br>3◯ 3<br>4◯ 4 Moderately focused<br>5◯ 5<br>6◯ 6<br>7◯ 7 Very unfocused/distracted
How irritable or easily angered do you feel right now?	1◯ 1 Not at all irritable/angry<br>2◯ 2<br>3◯ 3<br>4◯ 4 Moderately irritable<br>5◯ 5<br>6◯ 6<br>7◯ 7 Very irritable/angry
How quick is your thinking?	1◯ 1 Very quick/lots of ideas<br>2◯ 2<br>3◯ 3<br>4◯ 4 Neither of both<br>5◯ 5<br>6◯ 6<br>7◯ 7 Slow/cannot think of things
How much are you able to enjoy and feel pleasure in things?	1◯ 1 Really enjoying things<br>2◯ 2<br>3◯ 3<br>4◯ 4 Moderately enjoying things<br>5◯ 5<br>6◯ 6<br>7◯ 7 No pleasure or enjoyment

How fidgety or restless do you feel right now compared to your usual self?	1◯ 1 Not at all restless<br>2◯ 2<br>3◯ 3<br>4◯ 4 Moderately restless/fidgety<br>5◯ 5<br>6◯ 6<br>7◯ 7 Very restless/fidgety/cannot sit still
How hungry do you feel right now?	1◯ 1 Feeling full/not at all hungry<br>2◯ 2<br>3◯ 3<br>4◯ 4 Moderately hungry<br>5◯ 5<br>6◯ 6<br>7◯ 7 Extremely hungry
Since the last survey, I felt overwhelmed by a negative emotion.	1◯ 1 Not at all<br>2◯ 2<br>3◯ 3<br>4◯ 4 Moderately<br>5◯ 5<br>6◯ 6<br>7◯ 7 Yes, absolutely
Since the last questionnaire, I have lost control of myself.	1◯ 1 Not at all<br>2◯ 2<br>3◯ 3<br>4◯ 4 Moderately<br>5◯ 5<br>6◯ 6<br>7◯ 7 Yes, absolutely
**Physical activity**	
Please select the intensity level of activities you did since the last questionnaire:	1◯ Vigorous activities (e.g., running/fast cycling/heavy lifting or digging)<br>2◯ Moderate activities (e.g., tennis/bicycling/carrying light loads)<br>3◯ Light activities (e.g., walking/climbing stairs/routine household chores)<br>4◯ No physical activity at all.
Since you woke up, did you have a nap or rest?	1◯ Yes<br>2◯ No

**Drinks**	
Since the last survey, did you drink:	1☐ Water<br>2☐ Milk<br>3☐ A caffeinated beverage (like coffee/tea/soda etc.)<br>4☐ An alcoholic beverage (wine/beer/liquor etc.)<br>5☐ A beverage containing sugar (juice, ice-tea, or caffeine-free soda)<br>6☐ Another type of drink: _________<br>7☐ no drinks
[Only if yes to water]<br><br>How many 250 ml glasses of water did you consume?	__________
[Only if yes to milk]<br><br>How many 250 ml glasses of milk did you consume?	__________
[Only if yes to caffeinated beverage]<br><br>What type of caffeinated beverage did you consume?	1☐ Soda (Coke/Pepsi/other caffeinated soda)<br>2☐ Energy drink (Red Bull, etc.)<br>3☐ Coffee<br>4☐ Tea<br>5☐ Other: __________
[Only if yes to caffeinated beverage]<br><br>How many caffeinated drinks did you consume?	__________
[Only if yes to alcohol]<br><br>What type of alcoholic beverage did you consume?	1☐ Red wine<br>2☐ White wine<br>3☐ Champagne/sparkling wine<br>4☐ Beer<br>5☐ Cocktail<br>6☐ Whisky or other strong alcohol<br>7☐ Other type of alcoholic drink: __________
[Only if yes to alcohol]<br><br>How many servings of alcohol did you consume?	__________

[Only if yes to beverage containing sugar]<br><br>How many 250 ml glasses of high-sugar drinks did you consume?	___________
**Food**	
Since the last survey, how many times did you eat?	__________
[Only if yes]<br><br>What was the size of your meal?	1◯ A snack<br>2◯ A small meal<br>3◯ A regular/full meal<br>4◯ A large meal
[Only if yes to eating]<br><br>What was your biggest meal? The following questions are for this meal only.	1◯ A snack<br>2◯ A small meal<br>3◯ A regular/full meal<br>4◯ A large meal
[Only if yes to eating]<br><br>About what time did you eat your largest snack/meal?	Hour Minute<br>_____ : _____
[Only if yes to eating]<br><br>Please select all types of food you ate:	1☐ Beef/pork/lamb<br>2☐ Chicken or other poultry<br>3☐ Eggs<br>4☐ Fish<br>5☐ Dairy (yogurt/milk/cheese)<br>6☐ Bread/pasta/cereal/rice or other starchy food<br>7☐ Fruits<br>8☐ Vegetables<br>9☐ Sweet foods or candy<br>10☐ Energy bars<br>11☐ Potato chips or other salty snacks<br>12☐ Other: ___________

**Substances**	
Since the last survey you took last night and before this survey, have you used any of these substances?	Smoking/Vaping<br>1f☐/1v☐ Cigarette, e-cigarette<br>2f☐/2v☐ Cannabis/pot<br>3f☐/3v☐ CBD<br>4☐ Other drug<br>5☐ I did not use any substances.
[Only if yes to smoking]<br><br>How many cigarettes did you have?	___________
[Only if yes to e-cigarettes]<br><br>Since the last survey, on how many occasions have you been vaping e-cigarettes?	___________
[Only if yes to cannabis smoking]<br><br>How many joints (of cannabis/pot) did you have?	1◯ 1<br>2◯ 2<br>3◯ 3 or more
[Only if yes to cannabis vaping]<br><br>Since the last survey, on how many occasions have you been vaping cannabis, hashish, or other THC substances?	1◯ 1<br>2◯ 2<br>3◯ 3 or more
[Only if yes to CBD smoking]<br><br>How many CBD cigarettes have you smoked?	1◯ 1<br>2◯ 2<br>3◯ 3 or more
[Only if yes to CBD vaping]<br><br>Since the last survey, on how many occasions have you been vaping CBD?	1◯ 1<br>2◯ 2<br>3◯ 3 or more
[Only if yes to other drugs]<br><br>What other drugs did you use?	1☐ Cocaine<br>2☐ Tranquilizers<br>3☐ Stimulants<br>4☐ Heroin or other opiate<br>5☐ Other drugs: ___________

**Questions asked once in the evening**	
**Event of the day**	
In the last 24h, please think of the ONE event that affected you the most (positively or negatively), no matter how slightly.	
Which of the following categories best describes the area of your life in which the event occurred?	1◯ Education<br>2◯ Family or friend relationships<br>3◯ Interactions with peers<br>4◯ Interactions with strangers<br>5◯ Leisure<br>6◯ Exercise<br>7◯ Social media/networks<br>8◯ Health<br>9◯ Religion or spirituality<br>10◯ Traveling or commuting<br>11◯ Work<br>12◯ Other: ___________
To what degree did this event have a POSITIVE impact on you?	1◯ 1 No positive impact<br>2◯ 2<br>3◯ 3<br>4◯ 4<br>5◯ 5<br>6◯ 6<br>7◯ 7 Extremely positive
To what degree did this event have a NEGATIVE impact on you?	1◯ 1 No positive impact<br>2◯ 2<br>3◯ 3<br>4◯ 4<br>5◯ 5<br>6◯ 6<br>7◯ 7 Extremely positive
How did you react to this event?	
I talked about it with somebody.	Not at all<br>| Moderately<br>| Yes, a lot

I have thought about it a lot.	Not at all<br>| Moderately<br>| Yes, a lot
I reappraised it.	Not at all<br>| Moderately<br>| Yes, a lot
I just let it happen.	Not at all<br>| Moderately<br>| Yes, a lot
I expressed my emotions.	Not at all<br>| Moderately<br>| Yes, a lot
I tried to forget it quickly.	Not at all<br>| Moderately<br>| Yes, a lot
I tried to change the situation.	Not at all<br>| Moderately<br>| Yes, a lot

Social media

How much time in total over the past 24 hours have you used social media to view content (Instagram, YouTube, TikTok, Pinterest, Twitter, Snapchat, Facebook, etc.)?

Hours Minute
_____ : _____

How much time in total over the past 24 hours have you used social media to create content (Instagram, YouTube, TikTok, Pinterest, Twitter, Snapchat, Facebook, etc.)?

Hours Minute
_____ : _____

**Health**	
How was your physical health today?	Very poor<br>| Average<br>| Very good/excellent
Did you feel like you had no physical energy, as if you were weighted down or had a heavy feeling in your arms or legs for most of the day?	1◯ Yes<br>2◯ No
Did you take any over-the-counter medications today?	1◯ Yes<br>2◯ No
[Only if yes to medications]<br><br>Did you take them for:	1☐ Pain (headache/muscle/joint pain etc.)<br>2☐ Gastrointestinal/nausea/vomiting/bowel or stomach problems<br>3☐ Allergies/cold<br>4☐ Fever/acute illness<br>5☐ Headache<br>6☐ Sleep problems<br>7☐ Other: ___________
[Only if participant has already had her first menstrual periods]<br><br>Are you currently having your menstrual period?	1◯ Yes<br>2◯ No

Legend: to be noted that questions about alcohol consumption and substance use are not asked to preadolescents participants (groups 1 and 2). Additionally, adolescent participants (group 3) will respond four surveys a day, whereas preadolescents participants will only receive one survey in the morning and one in the evening.

Figure S1.

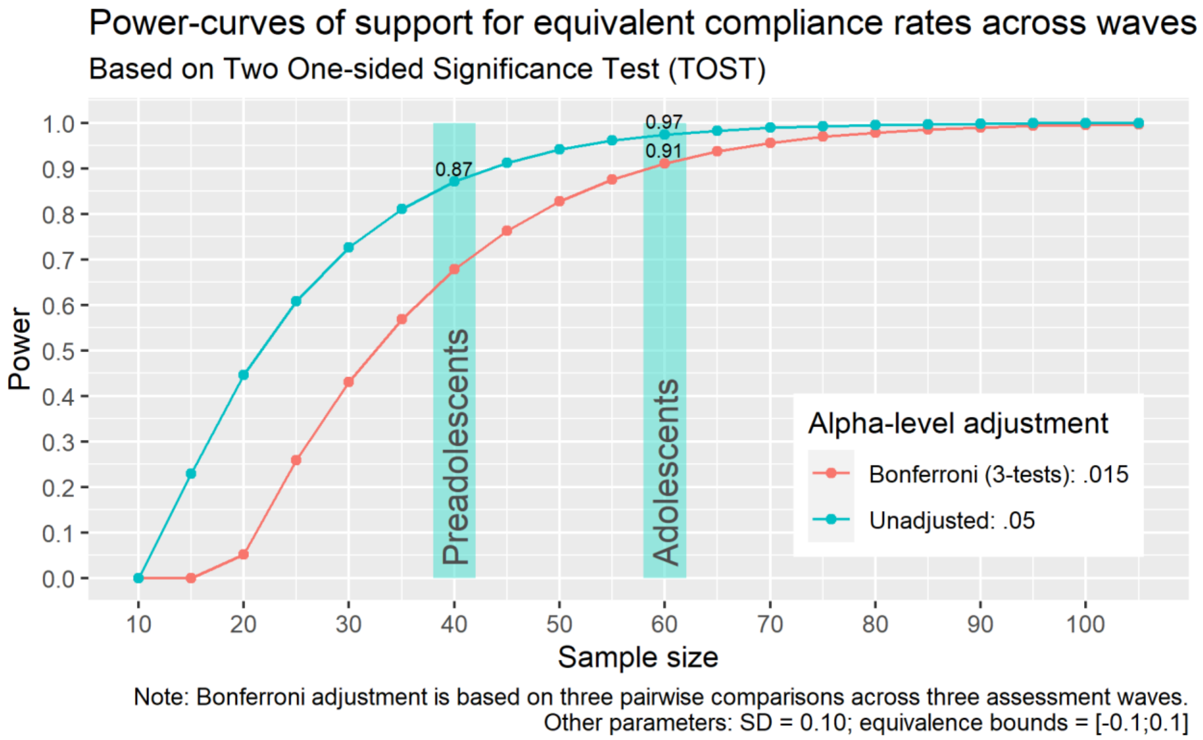
Power-curves of support for equivalent compliance rates across waves
Based on Two One-sided Significance Test (TOST)
Power
Sample size
0.87
0.97
0.91
Preadolescents
Adolescents
Alpha-level adjustment
Bonferroni (3-tests): .015
Unadjusted: .05
Note: Bonferroni adjustment is based on three pairwise comparisons across three assessment waves.
Other parameters: SD = 0.10; equivalence bounds = [-0.1;0.1]
